# Supplementary material for: Identification of Freezing-Responsive microRNAs and Their Targets in Chinese Jujube by Small RNA and Degradome Sequencing
Source: Int J Mol Sci. 2026 Sep 4;27(17):7912. doi: 10.3390/ijms27177912 (PMC13566890; doi:10.3390/ijms27177912)
Supplement: Supplementary file 1 [file ijms-27-07912-s001.zip › Supplementary Material S2/novel_miR_131.pdf]

Provisional ID	:	novel_miR_131
Score total	:	900.9
Score for star read(s)	:	3.9
Score for read counts	:	894.4
Score for mfe	:	1
Score for randfold	:	1.6
Score for cons. seed	:	
Total read count	:	1766
Mature read count	:	1748
Loop read count	:	0
Star read count	:	18

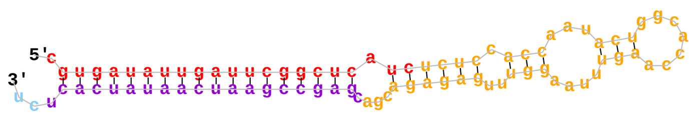

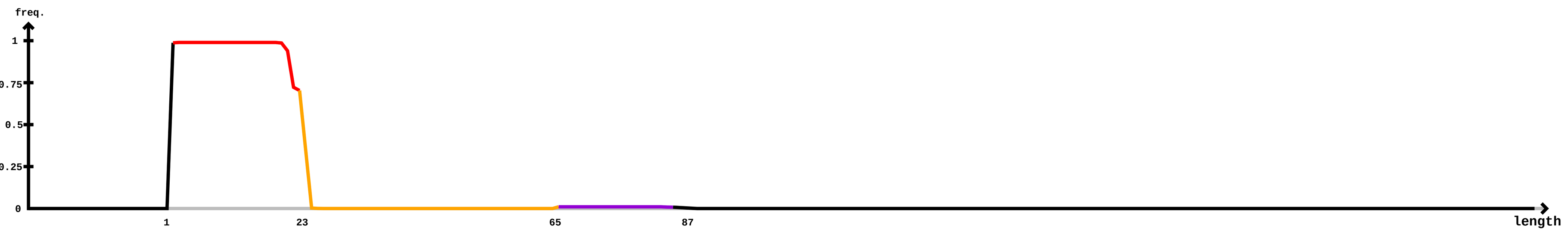

Mature Star

	reads	mm	sample
5'- cauucugcagaaguagacauggcgugauauugauucggcucaucucuccaccaauacuggcaccaaguuuaagguuugagagacgacgagccgaaucaauaucacucuuguaugcuucuucgcauauuaucaagguuuuuccuguuuuuugaguuccaauauuagccaagaagcaucucugcaggcaacucaugcauuaagcuacauagaagcuuguuuuggcucuccaucccccucuuguaaagucuuc -3' obs			
cauucugcagaaguagacauggcgugauauugauucggcucaucucuccaccaauacuggcaccaaguuuaagguuugagagacgacgagccgaaucaauaucacucuuguaugcuucuucgcauauuaucaagguuuuuccuguuuuuugaguuccaauauuagccaagaagcaucucugcaggcaacucaugcauuaagcuacauagaagcuuguuuuggcucuccaucccccucuuguaaagucuuc exp			
....((((((.........((.((((((((((((((((((.((((((.(((...(((.......)))....)))..))))))....))))))))))))))))).)).((((((......))))))......((.....))(((((((((.(((........))))))))))))..)))))))(((.....)))..((((((.......))))))....((((...((.........))...))))..	reads	mm	sample
......................cgugauauugauucggcu......	1	0	LT-1
......................cgugauauugauucggcu......	5	0	LT-3
......................cgugauauugauucggcuA......	6	1	LCK-3
......................cgugauauugauucggcuc......	7	0	LT-1
......................cgugauauugauucggcuU......	1	1	LT-3
......................cgugauauugauucggcuc......	21	0	LT-2
......................cgugCuauugauucggcuc......	1	1	LCK-3
......................cgugauauugauucggcuc......	13	0	LT-3
......................cgugauauugauucggcuc......	33	0	LCK-3
......................cgugauCuugauucggcuca......	1	1	LCK-1
......................cgugauauugauucggcuca......	23	0	LT-1
......................cgugauauugauucggcuca......	1	0	JT-2
......................cgugauauugauucggcuca......	2	0	JCK-1
......................cgugauauugauucggcuca......	60	0	LCK-2
......................cgugauauugauucggcuca......	9	0	JCK-2
......................cgugauauugauucggcuca......	3	0	JCK-3
......................cgugauauugauucggcuca......	103	0	LCK-3
......................cgugauauugauucggcuca......	3	0	JT-3
......................cgugauauugauucggcucU......	3	1	LT-2
......................cgugauCuugauucggcuca......	1	1	LT-3
......................cgugauauugauucggcuca......	48	0	LCK-1
......................cAugauauugauucggcuca......	1	1	LT-2
......................cgugauauugauucggcuca......	52	0	LT-3
......................cgugauCuugauucggcuca......	1	1	LCK-2
......................cgugauauugauucggcuca......	71	0	LT-2
......................cgugauauugauucgAcuca......	1	1	LCK-3
......................cgugauauugauucggcucau......	14	0	LCK-3
......................cgugauauugauucggcucaA......	1	1	LT-3
......................cgugauauugauucggcucau......	1	0	LT-3
......................cgugauauugauucggcucau......	8	0	LT-1
......................cgugauCuugauucggcucau......	1	1	LT-3
......................cgugauauugauucggcucau......	9	0	LT-2
......................cgugauauugauucggcucauc......	122	0	LT-2

Mature (cgugauauugauucggcucauc) — **Star** (cgagccgaaucaauaucacu)

cauucugcagaaguagacaugg**cgugauauugauucggcucauc**ucuccaccaauacuggcaccaaguuuaagguuugagagacga**cgagccgaaucaauaucacu**cuuguaugcuucuucgcauuaucaagguuuuccuguuuuugaguuccaauauuagccaagaagcaucucugcaggcaacucaugcauuaagcuacauagaagcuuguuuuggcucuccaucccccucuuguaaagucuuc

Agugauauugauucggcucauc	1	1	LT-1
cgugauauugauucggcucauc	248	0	LT-3
cgugauauugauucggcucauc	137	0	LCK-3
cNugauauugauucggcucauc	1	1	JCK-1
cgugauauugauucggcucauc	51	0	JCK-1
cgugCuauugauucggcucauc	1	1	LT-3
cgugauauugCuucggcucauc	1	1	JCK-2
cgugCuauugauucggcucauc	2	1	JCK-2
cgugauauugCuucggcucauc	1	1	LT-1
cgugauauugauucggcucauc	31	0	LCK-1
cguAauauugauucggcucauc	1	1	JCK-3
cgGgauauugauucggcucauc	1	1	LT-2
cgugauauugCuucggcucauc	1	1	JCK-1
cgugauauugauucggcucauc	99	0	JCK-2
cgugauauugauucggcucauc	93	0	JCK-3
cgugauauugauucggcucCuc	1	1	LT-2
cgugauauugauucggcucauc	237	0	LT-1
cguUauauugauucggcucauc	1	1	LT-2
cgugauauugauucggcucauU	13	1	LT-2
cgugauauugauucggcucauU	1	1	JCK-1
cgugauauugauucggcucauU	1	1	JCK-2
cgugauauugauucggcucauU	3	1	LT-1
cgugCuauugauucggcucauc	1	1	LCK-3
cgugauauugauucggcucauU	5	1	JCK-3
cgugauauugauucggcCcauc	1	1	LT-1
cgugauauugauucggcucauU	9	1	LCK-3
cgugauauugauucggcucauU	5	1	LT-3
cgugauauugauucggcucauc	31	0	LCK-2
cgugauauugauucggcucauUu	1	1	LT-3
cgugauauugauucggcucauUu	2	1	LT-1
cgugauauugauucggcucauUu	9	1	LCK-3
cgugauauugauucggcucaucA	2	1	LT-1
cgugauauugauucggcucaucu	15	0	LCK-3
cgugauauugauucggcucaucu	34	0	LT-3
cgugauauugauucggcucaucu	17	0	LT-2
cgugauauugauucggcucaucu	3	0	JCK-2
cgugauauugauucggcucaucA	1	1	LCK-3
cgugauauugauucggcucauAu	1	1	LT-2
cAugauauugauucggcucaucu	1	1	LT-2
cgugauauugauucggcucaucA	3	1	LT-2
cgugauauugCuucggcucaucu	1	1	LT-1
cgugauauugauucggcucaucu	4	0	JCK-3
cgugauauugauucggcucaucu	30	0	LT-1
cgugauauugauucggcucaucA	3	1	LT-3
cgugauauugauucggcucaucu	1	0	JCK-1
cgugauauugauucggcucauUu	6	1	LT-2
cgugauauugauucggcucaucuU	1	1	LT-2
cgugauauugauucggcucaucuU	2	1	LT-3
cgugauauugauucggcucaucuU	1	1	JCK-2
cgugauauugauucggcucaucuUu	1	1	LCK-3
gugauauugauucggcuca	1	0	LT-2
gugauauugauucggcucauc	2	0	LT-3
gugauauugauucggcucauc	1	0	LT-1
cgagccgaaucaauaucU	2	1	LCK-3
cgagccgaaucaauauca	1	0	LT-2
cgagccgaaucaauaucac	1	0	LT-1
cgagccgaaucaauaucacu	5	0	LT-2
cgagccgaaucaauaucacu	2	0	LT-3
cgagccgaaucaauaucacu	6	0	LT-1
cgagccgaaucaauaucacuU	1	1	LT-1
